# A strategy to optimize the thermoelectric performance in a spark plasma sintering process

**DOI:** 10.1038/srep23143

**Published:** 2016-03-15

**Authors:** Wan-Ting Chiu, Cheng-Lung Chen, Yang-Yuan Chen

**Affiliations:** 1 Institute of Physics, Academia Sinica, Taipei 11529, Taiwan; 2 Graduate Institute of Applied Physics, National Chengchi University, Taipei 11605, Taiwan

## Abstract

Spark plasma sintering (SPS) is currently widely applied to existing alloys as a means of further enhancing the alloys’ figure of merit. However, the determination of the optimal sintering condition is challenging in the SPS process. This report demonstrates a systematic way to independently optimize the Seebeck coefficient *S* and the ratio of electrical to thermal conductivity (σ/κ) and thus achieve the maximum figure of merit *zT* = S^2^(σ/κ)T. Sb_2−x_In_x_Te_3_ (x = 0–0.2) were chosen as examples to validate the method. Although high sintering temperature and pressure are helpful in enhancing the compactness and electrical conductivity of pressed samples, the resultant deteriorated Seebeck coefficient and increasing thermal conductivity eventually offset the benefit. We found that the optimal sintering temperature coincides with temperatures at which the maximum Seebeck coefficient begins to degrade, whereas the optimal sintering pressure coincided with the pressure at which the σ/κ ratio reaches a maximum. Based on this principle, the optimized sintering conditions were determined, and the *zT* of Sb_1.9_In_0.1_Te_3_ is raised to 0.92 at 600 K, showing an approximately 84% enhancement. This work develops a facile strategy for selecting the optimal SPS sintering condition to further enhance the *zT* of bulk specimens.

Thermoelectric (TE) materials have progressively attracted interest in recent years for their potential in our quest for a sustainable energy solution^1^. A measure of the performance of thermoelectric materials is the figure of merit: *zT* = σ*S*^2^T/κ, where *S* is the Seebeck coefficient, σ is the electrical conductivity, κ is the thermal conductivity, T is the absolute temperature, and the product (σ*S*^2^) is known as the power factor^2^. Specifically, a good TE material should have both a high Seebeck coefficient and a high σ/κ ratio. In the last decade, several strategies such as band structure engineering^3^, hierarchical architecture structuring^4^, and nanostructuring^5^ have been developed in efforts to overcome thermoelectric performance bottlenecks by reducing thermal conductivity and/or increasing the power factor to effectively enhance *zT*.

The thermoelectric performance of materials is highly sensitive to the materials’ microstructures and fabrication methods^6^. An ideal synthetic approach should produce thermoelectric materials with the following features: high and consistent quality, low cost, scalability, machinability, and good thermal and chemical stability. With regard to these qualities, the spark plasma sintering (SPS) technique has distinct advantages, which include rapid heating and electric current activation; the method also produces well-controlled microstructures. For these reasons, SPS has become a popular tool in thermoelectric research^7^,^8^,^9^. The SPS densification process is a temperature-dependent mass transfer process that entails a complex working mechanism. In brief, the sintering mechanism involves surface diffusion, evaporation, grain boundary diffusion and interparticle neck formation^10^,^11^. To achieve effective densification, the sintering parameters should be tuned to favour densification over coarsening, but elevated temperatures tend to cause particle coarsening and thereby adversely change the chemical composition and Seebeck coefficient of materials. The densification process is effectively a sliding and rearrangement of particles (or crystallites); therefore, with the assistance of an applied external pressure, the sintering temperature required for the densification can be significantly reduced.

Despite the fact that the SPS technique possesses great potential in creating high-performance composited bulk thermoelectric materials, very few studies have investigated the criterion for optimal sintering temperature and pressure in SPS processes. In this work, we offer a facile strategy in determining the optimal SPS sintering condition—specifically, by judging both the σ/κ ratio and the sintering temperatures at which the maximum Seebeck coefficient begins to degrade, thus proving the effectiveness of this strategy in the *zT* enhancement of bulk Sb_2−x_In_x_Te_3_ (x = 0–0.2) alloys. We also examined the applicability of our approach to other thermoelectric material systems and confirmed its generalisability to other systems that can be densified by the SPS process.

At 300–500 K, several potential thermoelectric materials are found in V–VI compounds such as Sb_2_Te_3_, Bi_2_Te_3_ and Bi_2_Se_3_^12^. To achieve higher *zT*, Bi-Sb-Te and Bi-Se-Te ternary alloys are developed by optimizing the carrier concentrations and thermal properties accordingly^13^. In contrast, Sb_2_Te_3_ attracts less attention because of its low figure of merit (*zT* ~ 0.3)^13^. Previous studies have indicated the presence of antisite defects in Sb-Te or Bi-Te alloys owing to their bond polarity similarity^14^. Because Sb-Te bond has relatively lower bond polarity compared with Bi-Te, the antisite defects are more severe in Sb-Te. More defects in the lattice certainly contribute extra carriers, too many of which inevitably lead to a poorer Seebeck coefficient and higher thermal conductivity^15^. Doping effects on Sb_2_Te_3_ have been intensively studied for theoretical and applicational purposes. Dopant candidates such as bismuth^16^, selenium^17^, titanium^18^, vanadium^19^, and indium^20^,^21^ have been examined. Indium is a promising dopant because it not only effectively suppresses the formation of antisite defects but also widens the bandgap of Sb_2_Te_3_, which is helpful in reducing the detrimental bipolar conduction at high temperatures. Under optimal SPS conditions, the In-doped Sb_2_Te_3_ alloys exhibited good thermoelectric performance with a *zT* value of approximately 0.73 at 600 K^22^.

In this study, *p*-type polycrystalline Sb_2−x_In_x_Te_3_ (x = 0, 0.05, 0.10, 0.15, 0.20) were prepared by melting and annealing, followed by the spark plasma sintering procedure. Six combinations of sintering temperature and pressure were performed on testing specimens of Sb_1.85_In_0.15_Te_3_ to identify the optimal sintering temperature and pressure by measuring their thermoelectric properties. The optimal SPS criterion was then applied to all other specimens to validate the methodology in engineering thermoelectric materials.

## Results and Discussion

### Spark plasma sintering condition optimization

**Figure 1:**
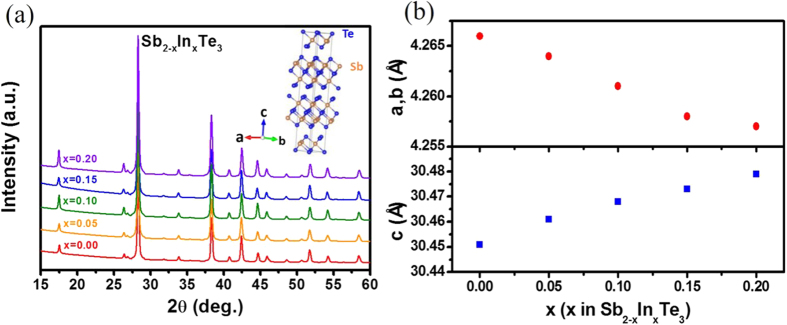
(**a**) Powder XRD patterns of Sb_2−x_In_x_Te_3_ (x = 0–0.2) samples, (**b**) lattice parameters as a function of x at 300 K. The inset of (**a**) shows the crystal structure of Sb_2_Te_3_.

Figure 1a shows the powder X-ray diffraction (XRD) patterns of Sb_2−x_In_x_Te_3_ with x = 0–0.2 grown by the method mentioned. The diffraction peaks can be indexed to the Sb_2_Te_3_ phase with a rhombohedral structure (JCPDS #15–0874), indicating Sb_2_Te_3_ phase, and no impurity phases were observed within the detection limit. The refined lattice parameters as a function of indium content are shown in Fig. 1b. The lattice parameter *a* and *b* decrease gradually with increasing indium concentrations, whereas the lattice parameter *c* shows the opposite trend, indicating that the lattices extend in *c* axis but shrink in the *ab* plane. The result is consistent with that reported by Rosenberg *et al*.^23^,^24^,^25^ and suggests that the added indium can successfully fill into the Sb site.

**Figure 2:**
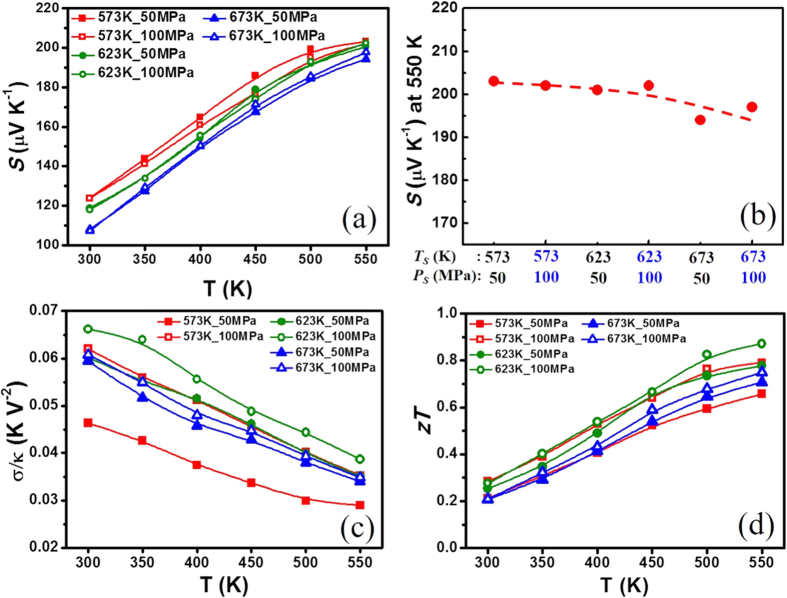
Temperature dependence of (**a**) Seebeck coefficient, (**b**) Seebeck coefficient at 550 K as a function of sintering temperature (*T*_*s*_) and pressure (*P*_*s*_), (**c**) σ/κ ratio, and (**d**) *zT* values for Sb_1.85_In_0.15_Te_3_ prepared by various SPS sintering conditions.

From the earlier work of In-doped alloys of Sb_2−x_In_x_Te_3_ by Hu *et al*.^22^, the alloy with x = 0.15 showed the best thermoelectric properties with maximum *zT* = 0.73 at 600 K. This doping level was therefore chosen for the experimental measurements to determine the optimal sintering temperature *T*_*S*_ and pressure *P*_*S*_ in the SPS process. Six combinations of sintering conditions were formed from the group of three temperatures—573, 623, and 673 K—and two pressures—50 and 100 MPa—for testing. As expected, the highest sintering temperature and pressure can achieve the highest mass density, which is near 100% of the theoretical value (6.5 g cm^−3^)^26^, but cannot guarantee the best thermoelectric performance (see Supplementary, Table S1). The underpinning drawback of higher sintering temperature is that it may induce chemical reactions that shift the ideal material composition away from that required by the optimal Seebeck coefficient^27^. This phenomenon indeed is observed, as shown in Fig. 2a, in which the peak Seebeck coefficient of approximately 203 μV K^−1^ (at T = 550 K) degraded to approximately 194 μV K^−1^ as the sintering temperature increased from 573 to 673 K. The plot of the Seebeck coefficient at 550 K as a function of the SPS parameters is given in Fig. 2b to further explain why the optimal sintering temperature coincides with temperatures at which the maximum Seebeck coefficient begins to degrade. Based on these analyses, the optimal sintering temperature is determined to be 623 K.

In addition to having a high Seebeck coefficient, a large σ/κ ratio is also required to achieve a higher *zT*. A high σ/κ ratio usually suggests excellent decoupling of carrier and phonon transport within the material. We found that achieving the highest electrical and thermal conductivity ratio σ/κ with the correct mass density is the best way to determine the sintering pressure. Figure 2c reveals that when the sample is sintered at 623 K with sintering pressure 100 MPa, the highest σ/κ ratio is obtained. The best sintering pressure is thus determined to be 100 MPa. As expected in Fig. 2d the highest *zT* = 0.85 at 550 K is obtained for the specimen sintered at 623 K/100 MPa. It is therefore noted that the highest ratio σ/κ is an essential criterion to obtain a high *zT* and is also an important guideline in determining the optimal SPS sintering pressure.

As a result, the optimal sintering condition of 623 K/100 MPa is chosen and then applied to other In-doped specimens. Sintering with longer holding times (10 min, or even 15 min) has also been examined in this sample system, but it has little effect on the thermoelectric properties of these samples. It can be understood that most of the grains are microsized; the effect of holding time on grain growth is almost ignorable. The sintering temperature and pressure are the main factors in improving the thermoelectric performance. We therefore maintained a holding time of 5 min in this study. To examine whether the proposed strategy can be applied to other thermoelectric material systems, two reported (Bi,Sb)_2_Te_3_ and PbTe material systems were examined accordingly^28^,^29^ (see Supplementary, Fig. S1). Indeed, our strategy does apply to their results.

**Figure 3:**
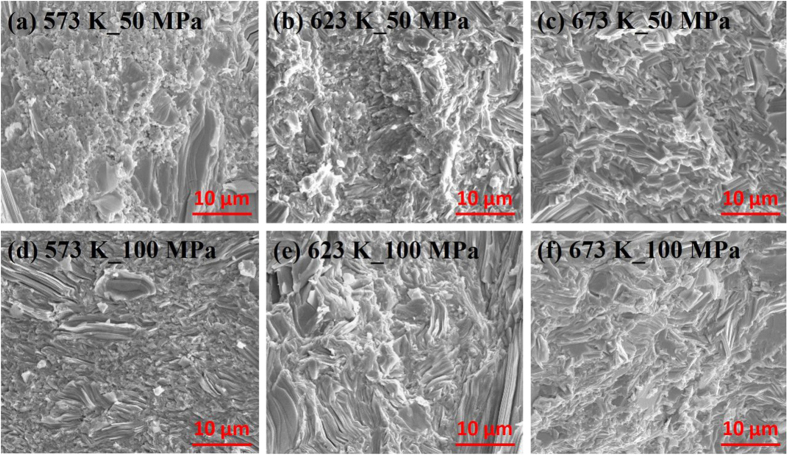
The SEM micrographs of fractured surface of samples prepared by various SPS conditions: (**a**) 573 K/50 MPa, (**b**) 623 K/50 MPa, (**c**) 673 K/50 MPa, (**d**) 573 K/100 MPa, (**e**) 623 K/100 MPa, and (**f**) 673 K/100 MPa. The images show the difference in the microstructures, and display the formation of platelet-like features.

Figure 3 shows the scanning electron micrographs of fractured surfaces of a series of samples under six different sintering conditions. A number of pores and small granule-like grains are observed in the sample densified with the lowest sintering temperature and pressure in Fig. 3a, which is also responsible for the lowest bulk density (approximately 91% of the theoretical density). By increasing the SPS temperature and pressure, the pores are almost eliminated, and the large grains show platelet-like structures with a tendency to align and stack together, which leads to a noticeably high bulk density and strong anisotropy (Fig. 3e,f). The average grain size of these samples is approximately 1–50 μm. The higher sintering pressure helps the particle sliding and rearrangement. The SEM results agree with the transport data and clearly reveal the enhanced compactness and bulk density as the sintering temperature and pressure increase.

**Figure 4:**
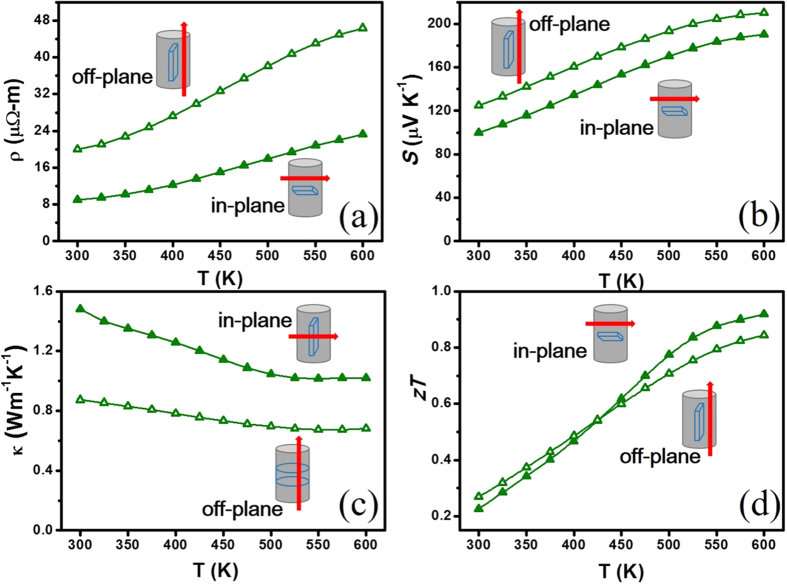
(**a**) Electrical resistivity, (**b**) Seebeck coefficients, (**c**) thermal conductivity, and (**d**) *zT* for two measurement directions for Sb_2−x_In_x_Te_3_ with x = 0.1. The schematic diagrams in (**a**–**d**) represent results measured from each slice of the cylinder.

The compositions of the phases and the distributions of elements of Sb_2−x_In_x_Te_3_ were analysed by an electron probe microanalyser (EPMA) (see Supplementary, Fig. S2). The elemental mapping of the indium-backscattered image shows a typical solid solution microstructure with no secondary-phase precipitates found in the sample. In the Sb and Te mapping results, although some Sb is substituted by Te in a small range of approximately 1 μm^2^, the distribution of the elements in the alloy is nearly homogeneous. The result agrees well with the liquidus projection of Sb-In-Te, and the secondary phase of In_2_Te_3_ will not precipitate until the indium content is greater than 45%^23^. The structural anisotropy phenomenon is observed in all SPS-sintering Sb_2−x_In_x_Te_3_ (x = 0–0.2) specimens (see Supplementary, Fig. S3). It is understood that Sb_2_Te_3_ is a layered structure material^30^, and the *ab* planes of the grains are easily reoriented into the disk plane during the sintering process. This anisotropic character is also reflected in the thermoelectric measurements as shown in the example specimen of Sb_1.9_In_0.1_Te_3_ (Fig. 4). The in-plane resistivity is approximately 55% lower than that of the off-plane direction (parallel to the hot-press direction) (Fig. 4a). Additionally, the Seebeck coefficient in-plane is approximately 20% lower than the other direction (Fig. 4b). A much lower thermal conductivity, as expected, in the off-plane direction is observed in Fig. 4c. Figure 4d shows that the in-plane *zT* result is better than that of the other direction.

### Thermoelectric properties

**Figure 5:**
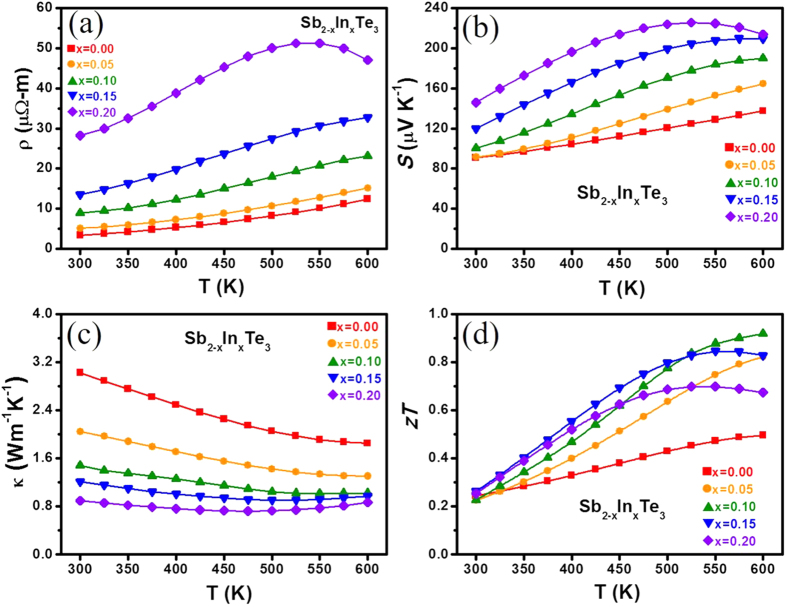
Temperature dependence of (**a**) electrical resistivity (**b**) Seebeck coefficient (**c**) thermal conductivity and (**d**) *zT* of Sb_2−x_In_x_Te_3_ for x = 0–0.2.

**Figure 6:**
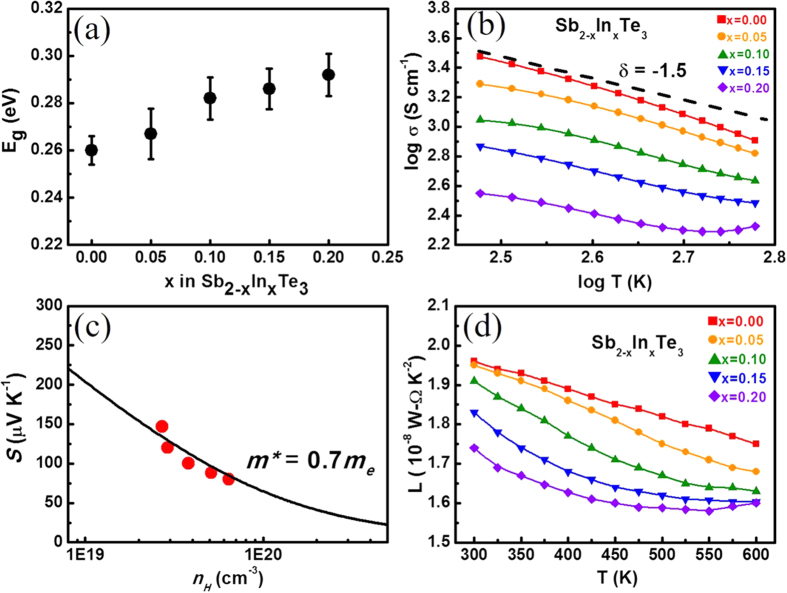
(**a**) Energy bandgaps, (**b**) log σ–log T plots—the line regime shows the slope δ = −1.5, (**c**) Seebeck coefficient as a function of Hall carrier density at 300 K, and (**d**) calculated temperature-dependent Lorenz number of Sb_2−x_In_x_Te_3_ (x = 0–0.2).

The temperature dependence of thermoelectric properties for Sb_2−x_In_x_Te_3_ with x = 0–0.2 are shown in Fig. 5. All measurements are along the direction perpendicular to the SPS press direction. The electrical resistivity increases with temperature for all samples, exhibiting a typical degenerate semiconductor behaviour (Fig. 5a). The resistivity increases significantly with increasing In content and can be attributed to the simultaneously decreased carrier density and mobility. The Hall measurement results for all samples at 300 K are summarized in Table 1. The temperature-dependent Seebeck coefficients are shown in Fig. 5b. It is well known that the binary Sb_2_Te_3_ has numerous inherent antisite defects in the crystal lattice, so a relatively high hole carrier density inevitably leads to a low Seebeck coefficient^16^. However, the gradual increase in Seebeck coefficient with increasing indium content is consistent with the decrease in Hall carrier density (Table 1). This suggests that In atoms substituting for Sb can properly reduce the hole carrier density by suppressing the antisite formation. From the Fourier transform infrared (FTIR) spectra and employing the Tauc plots^31^, the bandgaps (*E*_*g*_) of Sb_2−x_In_x_Te_3_ (x = 0–0.2) were determined (Fig. 6a, see Supplementary, Fig. S4). The *E*_*g*_ value of Sb_2_Te_3_ is approximately 0.26 eV, which is in good agreement with the literature data for the bulk Sb_2_Te_3_^32^. The bandgap widening of Sb_2_Te_3_ with increasing In content is shown in Fig. 6a and is probably responsible for the suppression of bipolar conduction at high temperatures. For all samples, the electrical conductivity decreases with increasing temperature, approximately following a *T*^−1.5^ relationship (Fig. 6b). The result implies that acoustic phonon scattering is responsible for the carrier scattering process^2^. Thus, the simple single parabolic band model with the acoustic phonon scattering assumption is utilized to analyse the variation of transport properties with In doping.

Figure 6c shows the relationship between Seebeck coefficient and Hall carrier density (Pisarenko relation) at 300 K and suggests a density of state effective mass *m*_d_* of approximately 0.7 *m*_*e*_, where *m*_d_* can be determined by the Hall carrier density (*n*_*H*_ = 1/*eR*_*H*_, *e* is the electron charge, and *R*_*H*_ is the measured Hall coefficient) via the following^33^:


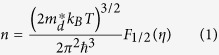



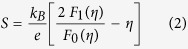



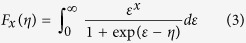


where *F*_*x*_(*η*) is the x-th order Fermi integral, and the Hall carrier density is related to the chemical carrier density *n* via *n*_*H *_= *n*/*r*_*H*_. *r*_*H*_ is the Hall factor for acoustic phonon scattering and is expressed as


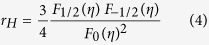


The power factors of Sb_2−x_In_x_Te_3_, except x = 0.2, show high values of 1.3 – 1.8 mW m^−1 ^K^−2^ over a temperature range of 450–600 K. Additionally, the power factors are not as sensitive to both indium content and temperature when T > 500 K. Thus, the additional reduction in thermal conductivity with increasing indium percentage is another crucial factor for *zT* enhancement. The temperature dependence of the thermal conductivity of Sb_2−x_In_x_Te_3_ with x = 0–0.2 is presented in Fig. 5c. Owing to the increased electrical resistivity and induced alloying scattering in In-doped samples, their thermal conductivities are significantly reduced over the whole temperature range. Moreover, the thermal conductivity of Sb_2−x_In_x_Te_3_ decreases gradually with increasing indium content and shows a slight increasing trend at elevated temperatures. Bipolar conduction may contribute here, and the detailed analysis will be discussed later.

The total thermal conductivity κ_tot_ is the sum of the electronic contribution κ_e_ and the lattice contribution κ_lat_. κ_e_ can be calculated by the Wiedemann–Franz law: κ_e_ = *L*σT, where *L*, σ and T are the Lorenz number, electrical conductivity and absolute temperature, respectively^34^. Usually, *L *= 2.45 × 10^−8 ^W–Ω K^−2^ is used for the free electron case. However, most good thermoelectric materials belong to heavily doped semiconductors, and a more reliable Lorenz number should be determined according to the reduced Fermi energy and scattering parameter (*r *= −1/2) as shown in the following equation^35^:


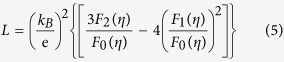


**Figure 7:**
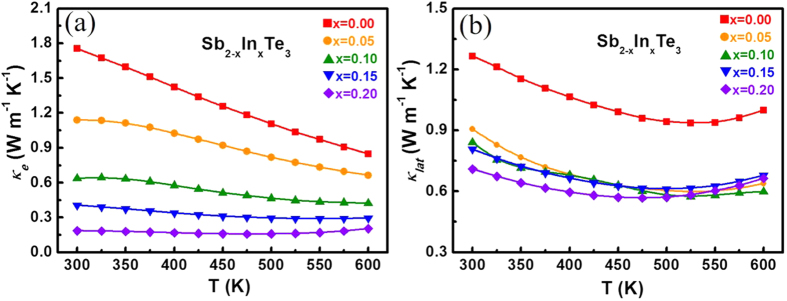
(**a**) Carrier thermal conductivity and (**b**) lattice thermal conductivity of Sb_2−x_In_x_Te_3_ (x = 0–0.2).

The *L* value for each indium doping content calculated by the equations above is presented in Fig. 6d. All values are lower than 2.45 × 10^−8 ^W–Ω K^−2^ and show a decreasing tendency with increasing temperature. The plot of κ_e_ versus temperature for all samples is presented in Fig. 7a. κ_lat_ is then calculated from κ_tot_ by subtracting κ_e_ (Fig. 7b). It is noted that κ_lat_ decreases rapidly with increasing indium doping owing to the enhanced alloy scatterings; however, the phonon scattering coming from the variation of In content in Sb_2_Te_3_ is quite limited. Moreover, κ_lat_ for all samples initially decreases with temperature but then increases when T > 500 K. This implies that the temperature is approaching the intrinsic excitation region^36^, and the bipolar diffusion may begin to contribute to the thermal conductivity.

*zT* as a function of temperature for all Sb_2−x_In_x_Te_3_ with x = 0–0.2 are shown in Fig. 5d. All In-doped specimens show a remarkable enhancement in *zT* values in the high temperature region. Sb_1.9_In_0.1_Te_3_ reaches a maximum *zT* of 0.92 at 600 K, showing an approximately 84% enhancement over the In-free sample. It even shows a 26% improvement compared with that reported by Hu *et al*.^22^. The significant *zT* enhancement can be attributed to the increased Seebeck coefficient and σ/κ ratio. However, a continued increase in the indium content does not further enhance *zT* owing to the rapid increase in electrical resistivity. An appropriate indium doping amount is required to maximize *zT* by suppressing the excessive carriers of Sb_2_Te_3_ and optimize the electrical and thermal conductivity. In addition, the reproducibility of SPS processing was examined from the four Sb_1.9_In_0.1_Te_3_ samples prepared with the same procedure. The narrow statistic distribution of *zT* values measured from these samples confirms the repeatability of the fabrication process for future applications (see Supplementary, Fig. S5).

## Conclusions

In summary, *p*-type polycrystalline Sb_2_Te_3_ with In doping was prepared by melting and annealing followed by spark plasma sintering. We demonstrated a systematic method to independently optimize the Seebeck coefficient *S* and the ratio of electrical to thermal conductivity (σ/κ) and thus achieve the maximum figure of merit *zT*. Temperature has the greater influence than pressure on the Seebeck coefficients during the sintering process. Sintering at 623 K/100 MPa is found to be the optimized condition for preparing Sb_2−x_In_x_Te_3_ samples with high σ/κ and Seebeck coefficient. The SPS-sintering Sb_2−x_In_x_Te_3_ samples show anisotropic behaviour in thermoelectric transport properties. A single parabolic band model with acoustic phonon scattering approximation is applied to characterize and explain all transport property data. The effective mass of Sb_2−x_In_x_Te_3_ alloys is estimated to be approximately 0.7 *m*_*e*_. The maximized *zT* value of the SPS-sintering Sb_1.9_In_0.1_Te_3_ specimen reaches 0.92 at 600 K, showing an approximately 84% enhancement over the pristine Sb_2_Te_3_. This work provides a more general and facile strategy for selecting the optimal SPS sintering condition to maximize *zT* of thermoelectric materials.

## Methods

Elements Sb, Te, and In, all of purity of 99.999%, were weighted with the appropriate molar ratio and sealed in a quartz tube that was evacuated to 10^−5^ mbar. It was kept in a furnace at 1023 K for 24 h and then quenched in cold water. The obtained ingots were further annealed at 723 K for 48 h. The annealed ingots were ground to powders with an agate mortar and then pressed using the spark plasma sintering (SPS-515S, SPS SYNTEX INC) method at 573–673 K and 50–100 MPa under vacuum for 5 min to form a dense pellet of 12.7 mm in diameter and 15 mm in height. The size allows electrical and thermal transport measurements in the same direction. The mass densities of samples can be greater than 95% of the bulk value. The grain size is estimated to be several micrometres. The temperature-dependent Seebeck coefficient and electrical conductivity were measured using a static dc method by a commercial system (ZEM-3, ULVAC-RIKO). The thermal conductivity was calculated from the relationship κ = λ ρ C_p_, where λ, ρ, and C_p_ are the thermal diffusivity, density, and heat capacity, respectively. The thermal diffusivity was measured by a laser flash apparatus (LFA-457, NETZSCH), and the heat capacity and thermogravimetry were measured by a differential scanning calorimeter (DSC-STA-449, NETZSCH). Mass density was measured by the method of Archimedes. All measured properties were acquired along the same direction. The uncertainty of each Seebeck coefficient, electrical resistivity and thermal conductivity measurements was estimated to be approximately 5%, which results in a total experimental uncertainty of *zT* of approximately 20%. The Hall effect measurements were measured in a magnetic field up to ±2T by a Physical Property Measurement System (PPMS, Quantum Design). The structural phase composition was analysed by X-ray diffraction, carried out with a diffractometer (XRD, PANalytical X’Pert Pro) equipped with Cu K_α_ radiation (0.154 nm). The microstructures of samples were investigated by scanning electron microscopy (SEM JXA-8200, JEOL). The compositions of the phase and the distribution of elements were identified by an electron probe microanalyser (EPMA JXA-8200, JEOL). The energy bandgaps of materials were obtained by infrared absorption measurements (Bruker Tensor 27 FTIR).

## Additional Information

**How to cite this article**: Chiu, W.-T. *et al*. A strategy to optimize the thermoelectric performance in a spark plasma sintering process. *Sci. Rep*. **6**, 23143; doi: 10.1038/srep23143 (2016).

## Supplementary Material

Supplementary Information

## Figures and Tables

**Table 1:** The mass density, electrical conductivity, Hall carrier density, Hall mobility, and Seebeck coefficient for the pressed Sb_2−x_In_x_Te_3_ (x = 0–0.2) at 300 K.

Sample compositions	*d*(g cm^−3^)	*σ*(S m^−1^)	*n*_*H*_(10^19^ cm^−3^)	*μ*(cm^2^ V^−1^ s^−1^)	*S*(μV K^−1^)
Sb_2_Te_3_	6.44	298507	6.42	291	90
Sb_1.95_In_0.05_Te_3_	6.35	194932	5.09	239	92
Sb_1.90_In_0.10_Te_3_	6.33	111349	3.82	182	100
Sb_1.85_In_0.15_Te_3_	6.30	73903	2.95	156	120
Sb_1.80_In_0.20_Te_3_	6.29	35433	2.67	83	146
