# Supplementary material for: A strategy to optimize the thermoelectric performance in a spark plasma sintering process
Source: Sci Rep. 2016 Mar 15;6:23143. doi: 10.1038/srep23143 (PMC4791595; doi:10.1038/srep23143)
Supplement: Supplementary Information [file srep23143-s1.pdf]

Supplementary Information

A strategy to optimize the thermoelectric performance in a spark plasma sintering process †

Wan-Ting Chiu[1], Cheng-Lung Chen[1*] & Yang-Yuan Chen[1,2*]

[1]Institute of Physics, Academia Sinica, Taipei 11529, Taiwan.

[2]Graduate Institute of Applied Physics, National Chengchi University, Taipei 11605, Taiwan.

* Corresponding authors

Table S1. The mass density (g cm^{-3}) of $Sb_{2-x}In_xTe_3$ (x=0–0.2) under different SPS sintering temperatures and pressures.

T (K) / P (MPa)	573 K	623 K	673 K
**50 MPa**	5.98	6.40	6.48
**100 MPa**	6.10	6.47	6.50

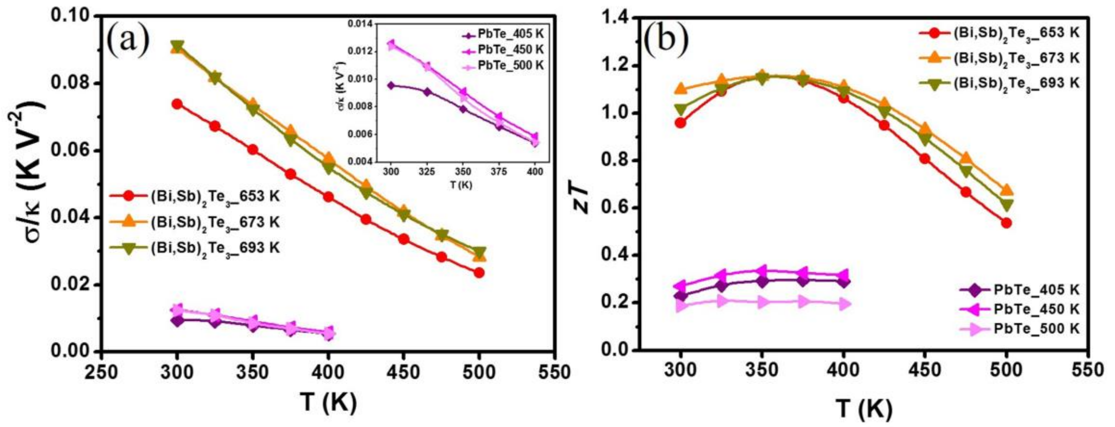

Figure S1. (a) Temperature dependence of σ/κ ratio and (b) Temperature dependence of *zT* for $(Bi,Sb)_2Te_3$ and PbTe[1,2].

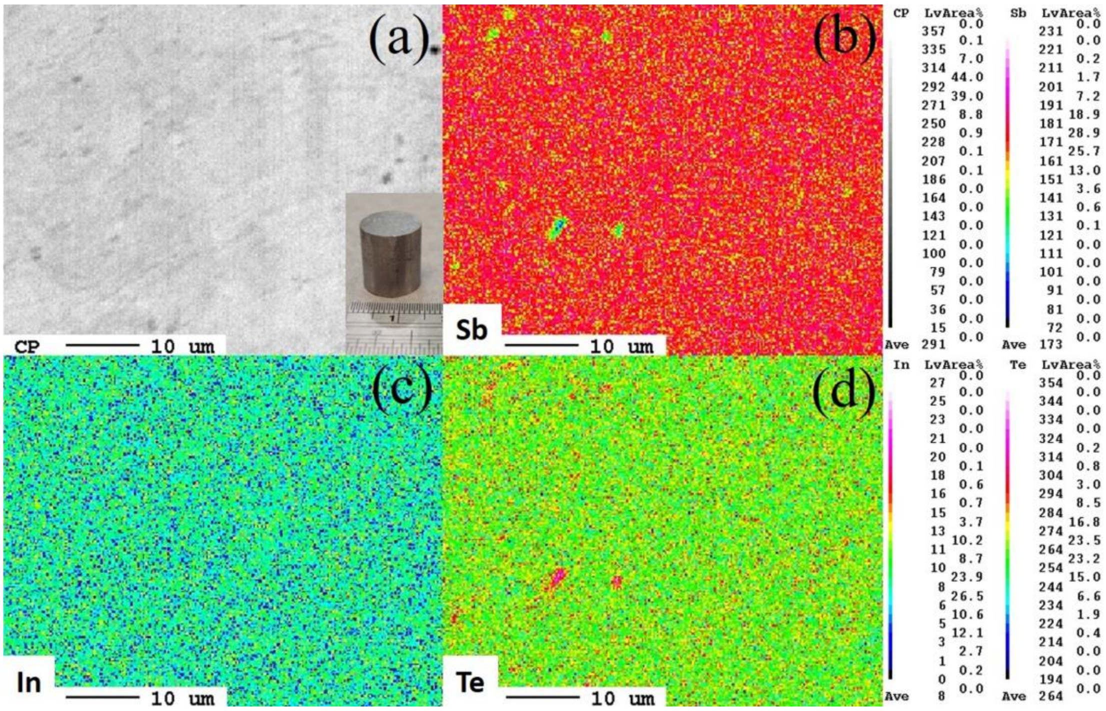

Figure S2. Microstructures and elemental mapping of $Sb_{1.90}In_{0.10}Te_3$ sintered at 623 K/ 100 MPa by SPS: (a) backscattered image at a large scale, (b) Sb mapping result, (c) In mapping result, and (d) Te mapping result. Inset image in (a): the hot pressed cylinder.

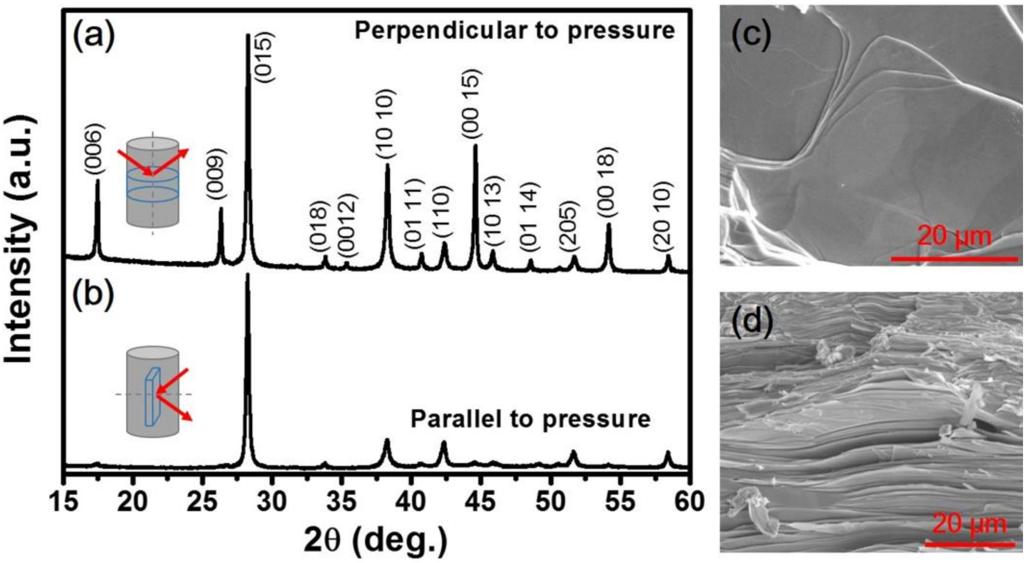

Figure S3. XRD patterns for the planes perpendicular (a) and parallel (b) to the press direction of the pressed $Sb_{1.90}In_{0.10}Te_3$ bulks sintered at 623 K/ 100 MPa. SEM images of the fractured surface of SPS-pressing samples in the direction (c) perpendicular and (d) parallel to the press direction.

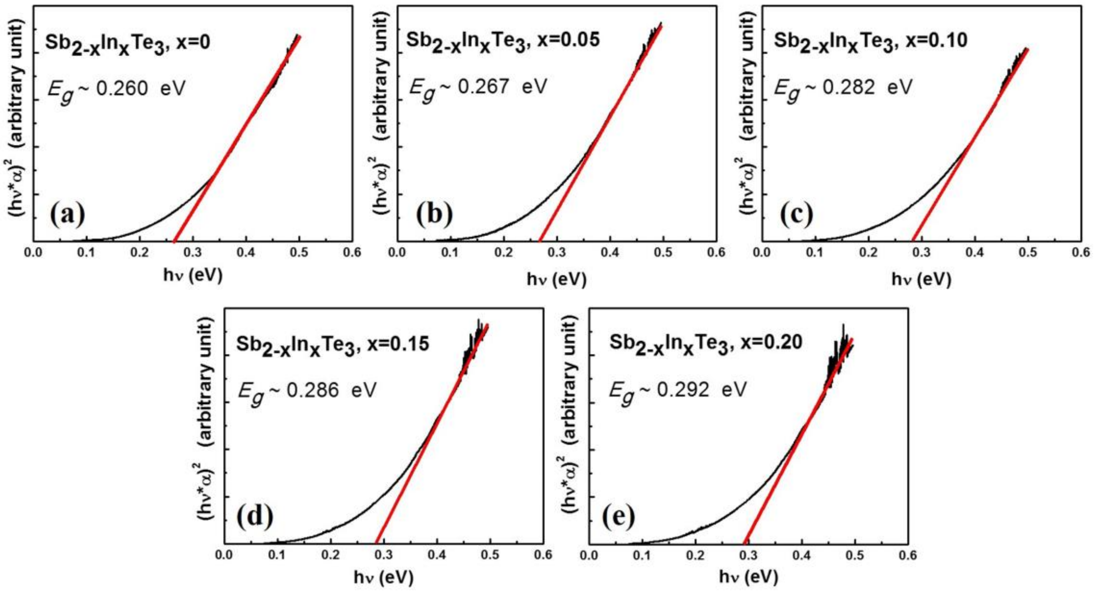

Figure S4. Plots of $(h\nu*\alpha)^2$ vs photon energy, hν (eV) for the $Sb_{2-x}In_xTe_3$ (x=0–0.2), where h is Planck's constant, ν is the frequency of photon, and α is absorption coefficient. (a) x=0, (b) x=0.05, (c) x=0.10, (d) x=0.15, and (e) x=0.20. The bandgap of each sample was determined by applying the Tauc relational expression[3].

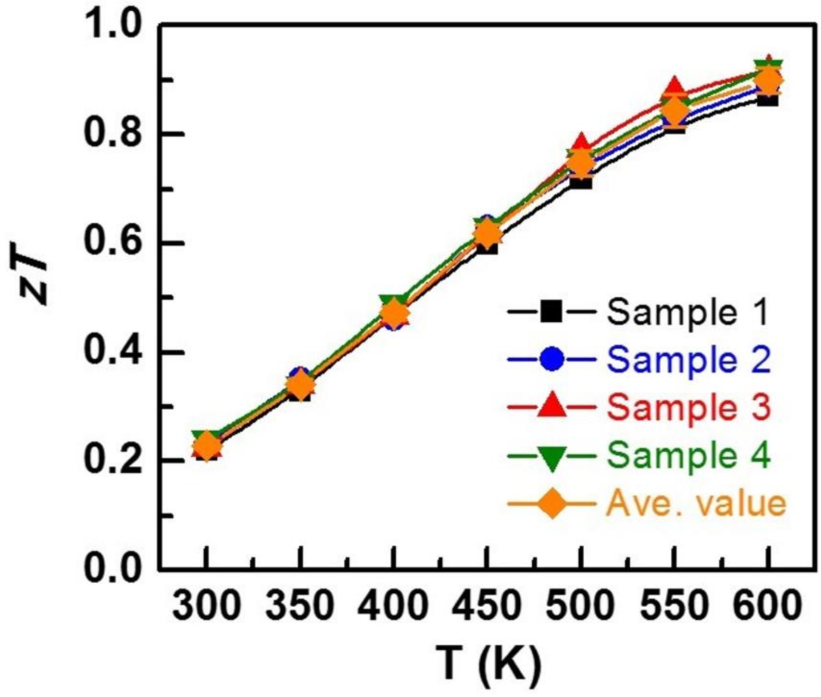

Figure S5. Reproducibility of $Sb_{1.90}In_{0.10}Te_3$.

Reference:

1. Jiang, J., Chen, L., Bai, S., Yao, Q. & Wang, Q. Thermoelectric properties of textured p-type $(Bi, Sb)_2Te_3$ fabricated by spark plasma sintering. *Scripta Mater.* **52**, 347-351 (2005).

2. Finefrock, S. W. *et al.* Structure and thermoelectric properties of spark plasma sintered ultrathin PbTe nanowires. *Nano Lett.* **14**, 3466-3473 (2014).

3. Tauc, J., Grigorovici, R. & Vancu, A. Optical properties and electronic structure of amorphous germanium. *Phys. Status Solidi (b)* **15**, 627-637 (1966).
